# StrainDesign: a comprehensive Python package for computational design of metabolic networks

**DOI:** 10.1093/bioinformatics/btac632

**Published:** 2022-09-16

**Authors:** Philipp Schneider, Pavlos Stephanos Bekiaris, Axel von Kamp, Steffen Klamt

**Affiliations:** Analysis and Redesign of Biological Networks, Max Planck Institute for Dynamics of Complex Technical Systems, Magdeburg 39106, Germany; Analysis and Redesign of Biological Networks, Max Planck Institute for Dynamics of Complex Technical Systems, Magdeburg 39106, Germany; Analysis and Redesign of Biological Networks, Max Planck Institute for Dynamics of Complex Technical Systems, Magdeburg 39106, Germany; Analysis and Redesign of Biological Networks, Max Planck Institute for Dynamics of Complex Technical Systems, Magdeburg 39106, Germany

## Abstract

**Summary:**

Various constraint-based optimization approaches have been developed for the computational analysis and design of metabolic networks. Herein, we present StrainDesign, a comprehensive Python package that builds upon the COBRApy toolbox and integrates the most popular metabolic design algorithms, including nested strain optimization methods such as OptKnock, RobustKnock and OptCouple as well as the more general minimal cut sets approach. The optimization approaches are embedded in individual modules, which can also be combined for setting up more elaborate strain design problems. Advanced features, such as the efficient integration of GPR rules and the possibility to consider gene and reaction additions or regulatory interventions, have been generalized and are available for all modules. The package uses state-of-the-art preprocessing methods, supports multiple solvers and provides a number of enhanced tools for analyzing computed intervention strategies including 2D and 3D plots of user-selected metabolic fluxes or yields. Furthermore, a user-friendly interface for the StrainDesign package has been implemented in the GUI-based metabolic modeling software CNApy. StrainDesign provides thus a unique and rich framework for computational strain design in Python, uniting many algorithmic developments in the field and allowing modular extension in the future.

**Availability and implementation:**

The StrainDesign package can be retrieved from PyPi, Anaconda and GitHub (https://github.com/klamt-lab/straindesign) and is also part of the latest CNApy package.

## 1 Introduction

Various constraint-based methods have been developed for the computer-aided design of metabolic networks and for the targeted optimization of microbial cell factories. As the first method of its kind, OptKnock (Burgard *et al.*, 2003) harnessed mixed-integer linear programming (MILP) for the rational design of microbial production hosts based on the concept of growth-coupled production. Today, a plethora of computational methods exists for various applications of metabolic design, for example, to enforce growth-coupled or even growth-independent production of a target chemical or to find synthetic lethals (Cardoso *et al.*, 2018; Jensen *et al.*, 2019; Pereira *et al.*, 2021; Schneider *et al.*, 2020, 2021; Tepper and Shlomi, 2010).

However, the landscape of toolboxes provided for strain design is very scattered, often hindering a broader and more efficient application of metabolic design approaches in combination with effective analysis tools. Despite the increasing number of constraint-based modeling tools developed in Python [e.g. ScrumPy (Poolman, 2006), COBRApy (Ebrahim *et al.*, 2013), cameo (Cardoso *et al.*, 2018), OptCouple (Jensen *et al.*, 2019), ReFramed (https://zenodo.org/record/4700490), CNApy (Thiele *et al.*, 2021) and MEWpy (Pereira *et al.*, 2021)], available packages with strain design algorithms are still distributed over different platforms including the Python packages mentioned above, the MATLAB-based COBRA toolbox (Heirendt *et al.*, 2019) and CellNetAnalyzer (von Kamp *et al.*, 2017), as well as the Java-based OptFlux (Rocha *et al.*, 2010). Moreover, each of these packages focuses only on one or very few of the published design methods. In particular, no toolbox exists that provides bi-level optimization techniques (e.g. OptKnock, RobustKnock and OptCouple) together with the more general minimal cut set (MCS) approach (Schneider *et al.*, 2020, 2021). A single framework allowing the use (and comparison) or even combination of different classes of methods would be highly desirable. This would also facilitate the reuse of modules developed for certain subtasks relevant for all optimization approaches (e.g. preprocessing and network compression).

Herein, we present the Python package StrainDesign, which aims to provide an integrated and extendable framework for MILP-based strain optimization approaches. StrainDesign also offers tools for the efficient analysis of calculated design strategies and a developed interface for CNApy provides the option to access most features and methods of the package via a user-friendly graphical environment.

## 2 Features and implementation

### 2.1 Strain design algorithms

**Fig. 1.**
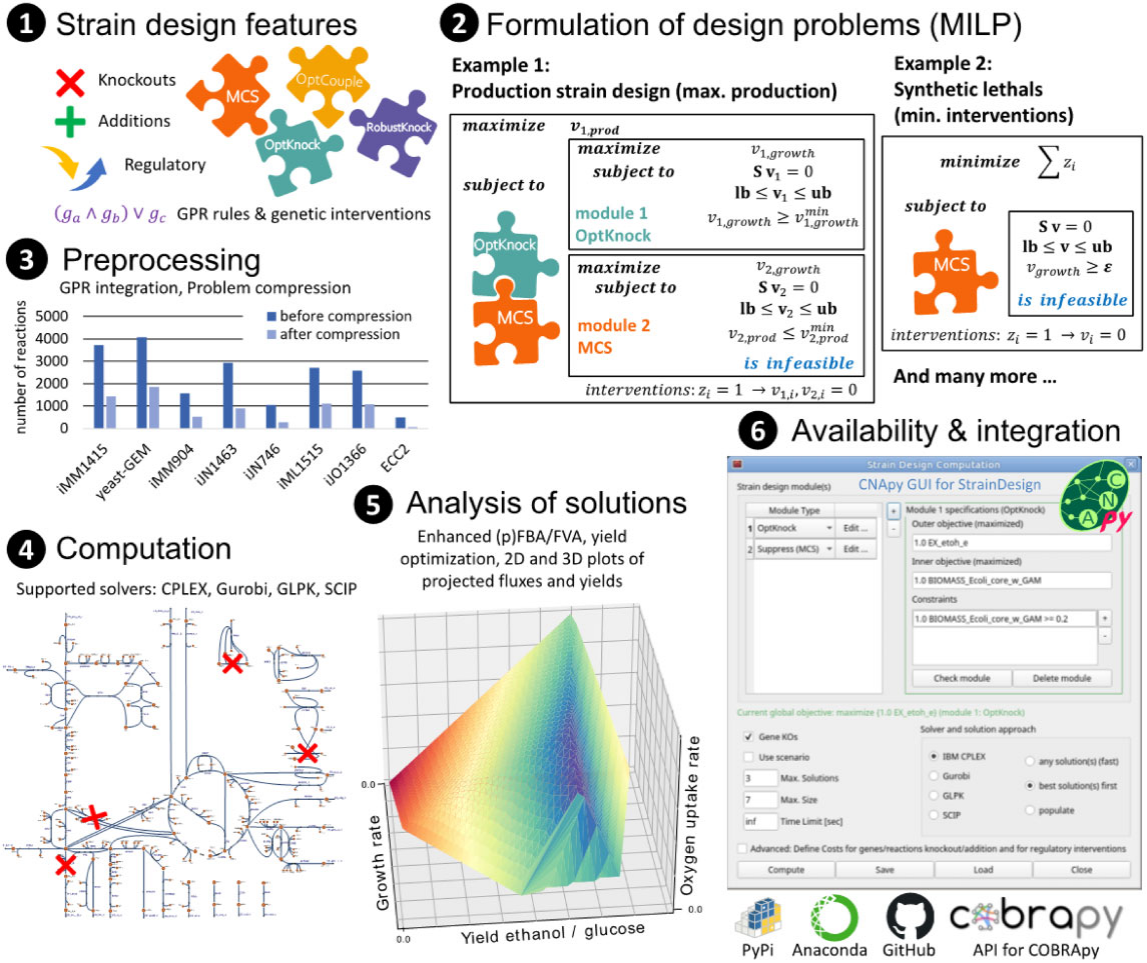
Overview of features and workflows of the StrainDesign package. For further explanations, see text

The StrainDesign package integrates currently four of the most popular MILP-based strain design approaches RobustKnock (Tepper and Shlomi, 2010), OptCouple (Jensen *et al.*, 2019), OptKnock (Burgard *et al.*, 2003) and MCS (Schneider *et al.*, 2020, 2021). The specification of the respective design problems is simplified by predefined optimization modules; it requires only minimal user input (e.g. definition of the objective function) and all steps for the MILP construction are automated. Moreover, StrainDesign allows combinations of protected (desired) and suppressed (undesired) regions, known from MCS-related formulations (Schneider *et al.*, 2020), with one of the other three (multi-level) MILP problems. One such hybrid approach is shown in the first example under point (2) in Figure 1. This example uses an OptKnock module to maximize the possible production rate of a desired chemical at maximum growth. At the same time, the added MCS-like module suppresses knockout sets that would lead to low production rates at maximum growth. In contrast to classical OptKnock solutions, this formulation guarantees growth-coupled production. The corresponding design objective function (here: maximization of product synthesis) is taken from the selected bi-level optimization module OptKnock. The second example in Figure 1 shows the computation of synthetic lethals, which can be formulated as a pure MCS problem, where the number of knockouts is minimized to obtain a non-viable phenotype.

There are various features, options and parameters that can be used for the specification of design problems, for example, allowance of interventions on reaction or gene level, regulatory interventions, reaction (or) gene additions, number of solutions to be calculated, the maximal number of allowed interventions, allowance of sub-optimal (e.g. non-minimal) solutions to speed-up calculations and many others.

The computational pipeline is largely standardized for all design problems and involves a comprehensive preprocessing routine. As one building block, if the user demands to compute intervention sets operating on genes, gene–protein-reaction associations are compressed and integrated into the metabolic network structure as described in Schneider *et al.* (2020). Regulatory interventions can be emulated by introducing additional variables and constraints in the form of pseudo-reactions and metabolites (Mahadevan *et al.*, 2015). Likewise, potential reaction (or gene) additions are treated as inverse knockouts in the MILP (Schneider *et al.*, 2020). Powerful network compression techniques, adopted and partially extended from efmtool (Terzer and Stelling, 2008), are applied to the entire model structure to reduce the problem size. Flux variability analysis (FVA) is used to identify reactions being essential for protected phenotypes, which are then excluded as possible intervention targets to further reduce the search space.

With its supported features, StrainDesign can emulate several other computational strain design approaches including gMCS (Apaolaza *et al.*, 2019), cRegMCS (Mahadevan *et al.*, 2015), FOCAL (Tervo and Reed, 2012), OptKnock with tilted objective function (Feist *et al.*, 2010) and ModCell2 (Garcia and Trinh, 2019).

### 2.2 Strain analysis tools

A collection of canonical linear-programming-based analysis tools complements the actual design algorithm. These tools are, on the one hand, useful to analyze a wild type’s metabolism (e.g. maximum biomass and product yields, production envelopes etc.) and to formulate, from this information, realistic strain design goals. On the other hand, these analysis tools are indispensable for validating and evaluating computed strain designs regarding their fitness and production capacities. Analysis methods integrated in the StrainDesign package include flux balance analysis (FBA), FVA, true yield optimization through linear-fractional programming (Klamt *et al.*, 2018) and plotting functions to generate arbitrary 2D or even 3D projections of the metabolic flux space onto user-selected reaction rates or yield terms (including 2D and 3D production envelopes and yield spaces; see example in Fig. 1). StrainDesign’s FBA and FVA implementations also support SCIP and, for FVA, parallelized computations required for efficient preprocessing and analysis of large (genome-scale) models.

### 2.3 Implementation, integration in CNApy and availability

The StrainDesign Python package operates on COBRApy models. Since COBRApy ships with the open-source MILP solver GLPK, no further packages are required for small-scale computations. The more powerful MILP solvers IBM CPLEX, Gurobi and the open-source alternative SCIP (Bestuzheva *et al.*, 2021) are also supported by StrainDesign. The ability to use indicator constraints by these solvers often avoids numerical problems and increases the speed of the computations.

Importantly, all functions from the StrainDesign package can also be accessed through a graphical user interface developed and integrated in the metabolic modeling package CNApy (Thiele *et al.*, 2021) (see the screenshot of the dialog box in Fig. 1). This enables also non-experts to use all features of StrainDesign in a user-friendly environment. Moreover, the computed intervention strategies can be displayed and analyzed within a network visualization.

StrainDesign can be retrieved from the package indexes PyPi and Anaconda. Source code, examples and documentation are available on GitHub (https://github.com/klamt-lab/straindesign). The latest CNApy package (release 1.1.1) contains the GUI interface for StrainDesign.

## 3 Conclusion

StrainDesign is a Python package that builds upon the COBRApy-framework and provides a single platform with a large collection of advanced methods and features for MILP-based metabolic network design and strain optimization. In particular, it is the first Python package supporting the powerful and very flexible MCS framework, which can now also be combined (or compared) with multi-level (nested) optimization approaches. Together with the different supported intervention types, a huge variety of design problems can be formulated and state-of-the-art network compression techniques allow their efficient treatment also in large-scale networks. Regarding MEWpy and cameo, two existing Python-based packages for metabolic network design, our toolbox complements MEWpy, which focuses on evolutionary algorithms for strain design, and it has only a small overlap (OptKnock) with cameo. The StrainDesign toolbox can be used in Python scripts or programs or accessed via the GUI of CNApy. The integration of further strain optimization techniques in the future is facilitated by a modular structure of the computational pipeline.

## Author contributions

P.S.: investigation, software (main developer), writing (original draft, review and editing). P.S.B.: software (integration of StrainDesign in CNApy, testing), writing (review and editing). A.V.K.: software (efmtool interface and testing), writing (review). S.K.: Supervision, funding acquisition, software (testing), writing (review and editing).

## Funding

This work was supported by the German Federal Ministry of Education and Research [de.NBI partner project ‘ModSim’ (FKZ: 031L104B)] and by the European Research Council [721176].

*Conflict of Interest*: none declared.
